# Audio and linguistic prediction of objective and subjective cognition in older adults: what is the role of different prompts?

**DOI:** 10.3389/fpsyt.2025.1596132

**Published:** 2025-07-01

**Authors:** Varsha D. Badal, Caitlyn Tran, Haze Brown, Danielle K. Glorioso, Rebecca Daly, Anthony J. A. Molina, Alison A. Moore, Erhan Bilal, Ellen E. Lee, Colin A. Depp

**Affiliations:** ^1^ Department of Psychiatry, University of California San Diego, San Diego, CA, United States; ^2^ Stein Institute for Research on Aging, University of California San Diego, San Diego, CA, United States; ^3^ University of California San Diego, San Diego, CA, United States; ^4^ Department of Medicine, University of California San Diego, La Jolla, CA, United States; ^5^ International Business Machines Corporation (IBM) Research, Yorktown, NY, United States; ^6^ Veterans Affairs (VA) San Diego Healthcare System, La Jolla, CA, United States

**Keywords:** acoustic, psycholinguistic, cognitive impairment, dementia, machine learning, NLP, Alzheimer’s

## Abstract

**Background:**

Psycho-linguistic and audio data derived from speech may be useful in screening and monitoring cognitive aging. However, there are gaps in understanding the predictive value of different prompts (e.g., open ended or structured) and the relationship of features to subjective versus objective cognition.

**Objective:**

To advance understanding of method variation in speech-analysis based psychometry, we evaluated targeted prompts for classification of impaired cognition and cognitive complaints.

**Method:**

A sample of 49 older participants (mean age: 76.9, SD: 8.5) completed short interview questions and cognitive assessments. Acoustic and Linguistic Inquiry through Word Counting i.e., LIWC (verbal content-based) features were derived from answers to open ended questions about aging (AG) and the Cookie Theft task (CT). Outcomes were objective cognitive ability measured using Telephone Interview for Cognitive Status (TICS-m), and subjective cognition using Cognitive Failures Questionnaire (CFQ).

**Results:**

A combined feature set including acoustic and LIWC (verbal content) yielded excellent classification results for both CFQ and TICS-m. The F1, precision and recall for CFQ elevation was 0.83, 0.85 and 0.82, and for TICS-m cutoff was 0.92, 0.92 and 0.92 respectively (using single learners). Features derived from CT task were of greater relevance to TICS-m classification, while the features from the AG task were of greater relevance to the CFQ classification.

**Conclusion:**

Acoustic and psycholinguistic features are relevant to assessment of cognition and subjective cognitive complaints, with combined features performing best. However, subjective and objective cognitions were predicted to differing extents by the different tasks, and the feature sets.

## 1 Introduction

It is well established that age-related cognitive decline co-occurs with changes detectable in speech (1). Changes in speech appear also to be associated with risk of Alzheimer’s disease (2). Speech analysis has primarily derived from samples of responses to structured prompts, but audio and psycho-linguistic analysis of verbal responses in open ended conversation may also predict objective cognitive performance (3). In addition to prediction of objective cognitive impairments, the application of speech analysis to prediction about subjective cognitive complaints or SCC (e.g. concerns about memory or slow thinking) has received little study (4). SCC are a key component of screening and diagnosis of Mild Cognitive Impairment (MCI). SCC remain unexplored through automatic voice and speech-analysis based techniques, leaving a gap in how speech task variants correlate with subjective cognition.

Although both relevant and common, SCC (5–9) are distinct from cognitive impairments (10). SCC is positively correlated with depressive symptoms (11), more so than are objective measures of cognition (12–15). Identifying which features from audio-based samples predict subjective, objective cognition, and both, could be helpful in understanding the potential utility of speech analysis.

The type of conducted dialogue (e.g., unstructured interviews vs. directed instructions) and the topic may influence not only the sentiment and non-verbal vocalizations, but also the content and framing of responses (16–18). Cognitive impairment has been explored through automated speech analysis using several kinds of dialogues with humans or software agents. Some of these dialogues are everyday conversation with humanoid robot (19), computer avatar based conversations (20), casual conversation (21–23), story retelling (24), recalling content of film (25), picture description task (26) and directed questions such as birthplace, name of elementary school, time orientation and backward recitation of three digit numbers (27). Among these, the Cookie Theft task (28, 29), which is a directed picture description task, has been a popular choice (30–34). To our knowledge, few or no studies have evaluated differences in prediction from different speech data sources within the same sample.

Recorded conversational speech offers a variety of features: acoustic, linguistic and verbal content; each offering a different insight (18). These layers often intertwine and influence each other; for example, a speaker’s voice acoustics may betray underlying emotions that can significantly impact the interpretation of the information (content) as well as speaker’s age and health. Acoustic feature sets are often large, openSMILE (35–37) comprises a set of 88 features, some of which have been related to psychological processes. In speech analysis, “shimmer” is a measured acoustic feature that quantifies the cycle-to-cycle variation in the amplitude (volume) of a voice signal, as to how much the loudness fluctuates between each vocal fold vibration. Shimmer features studies (38, 39) suggest a link with emotions, and as indicators of cognition decline (40). More recently, formant frequencies were shown to undergo a predictable change under cognitive load (41). Linguistic Inquiry and Word Count (LIWC) (42) counts words which are assigned into various psychological and linguistic categories (43). Speech transcribed through text can be effectively processed using LIWC for content analysis (44) for mood (45) as well as objective cognition (46) and other constructs (47). Although we could find no studies on subjective cognition using LIWC, it is a strong candidate feature set for such analyses.

A recent comprehensive review of NLP and audio based studies on detection of cognitive impairment (48) summarized that most prior work included both NLP and audio analyses in the same sample. Among studies reviewed, speech elicitation methods varied from spontaneous speech, clinical interviews, and conversations with virtual agents. The analysis for the CT task mostly relied upon NLP based techniques using n-grams, BERT-embeddings, Transformer encodings, GPT encodings; only three combined both NLP and acoustic features. Another review focused only on automated speech recognition based methods (49) included only three studies combining NLP and audio features, one included immediate and delayed recall of a short film and two using cookie theft task. Techniques combing NLP and speech performed generally better than either one separately. No study to our knowledge addressed both objective cognition and subjective cognitive complaints while combining NLP and audio features.

In this study, we contrast two approaches to speech data collection: the Cookie theft picture description task that invokes cognitive processing, and the other more open-ended prompting to describe individual experiences of aging. The choice of prompts (Cookie Theft Task, successful aging questions) in this pilot study were partially dictated by the prevalent norms, especially the cookie theft prompt. The Cookie Theft Task (28) has a long history in aphasia diagnosis (29), with as well as in research on Alzheimer’s (50). The task was also selected because it has been extensively evaluated using speech analysis (31, 48, 49, 51, 52). The aging questions were chosen as a complementary task because they were previously evaluated in other studies of healthy aging (53) and has been subjected to linguistic and voice analyses (3, 54, 55), Finally, both the Cookie Theft task and successful aging questions can be delivered by remote means making them scalable.

We also evaluated the association of these modalities with differential prediction of subjective cognitive ability and objective impairment. We hypothesized (based on prior literature) that both speech elicitation prompts would yield data that would result in reasonable levels of accuracy in discriminating individuals above the cutoffs from those below, for subjective as well as objective measures. We also hypothesized that acoustic and linguistic features could attain good performance (F1>0.75) in predicting both subjective and objective cognitive abilities. We explored the variation in contribution of acoustic versus linguistic features to integrated models, and then contrasted features derived from the different speech elicitation methods in predicting subjective versus objective cognitive impairments.

## 2 Materials and methods

### 2.1 Participants and procedures

The participants were drawn from a previously engaged large sample of 1300 community -dwelling residents of San Diego County for the parent study, the Successful AGing Evaluation (SAGE) (56). That project is detailed elsewhere, and briefly, used random digit dialing to recruit a sample of 1006 persons. Participants completed a baseline assessment consisting of a set of survey instruments and thereafter participants were followed on an annual basis with some exceptional years. A subsample (n=311) that had expressed interest in future studies on aging were contacted via mail with a pamphlet describing the goals of this study.

We augmented the SAGE survey (56) to include brief telephone or Zoom interviews of SAGE participants. The SAGE study (56) had the following inclusion criteria: (1) age 50–99 years, (2) having a (landline) telephone at home, (3) physical and mental ability to participate in a telephone interview and to complete a paper and pencil mail survey, (4) informed consent for study participation, and (5) English fluency and the exclusion criteria w: (1) residence in a nursing home, or requiring daily skilled nursing care, and (2) self-reported prior diagnosis of dementia, (3) terminal illness, or requiring hospice care. The study protocol was approved by the IRB of the University of California San Diego.

**Figure 1:**
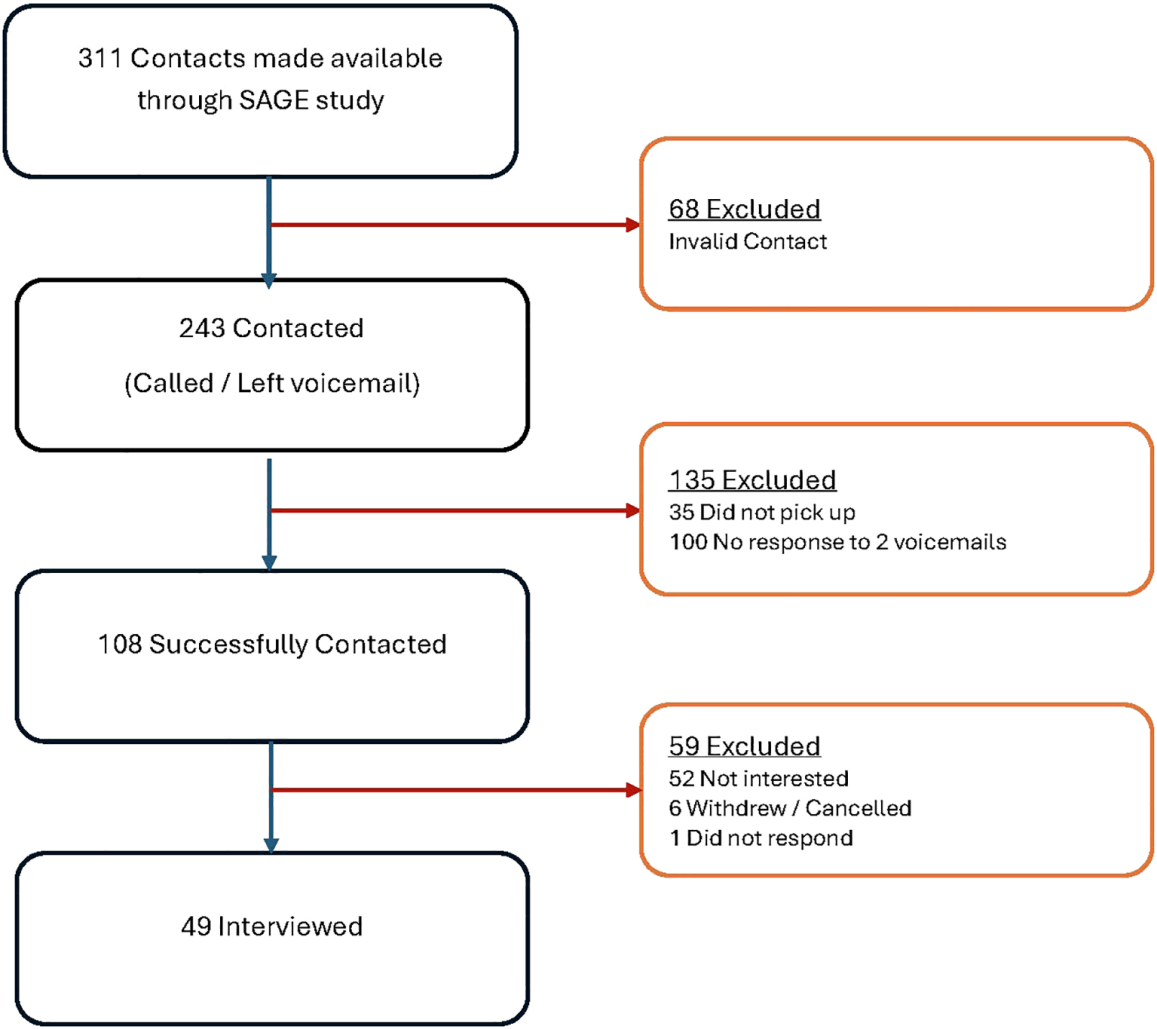
Flowchart of participant retention through the study.

A subsample (n=311) that had expressed interest in future studies on aging were contacted via mail with a pamphlet describing the goals of this study of which 49 participated in the study. The severe attrition of the sample was attributed to several reasons, some of which are detailed as follows (**Figure 1**). Some of the phones were out of service or incorrect numbers (68), some did not pick up the phone and no voicemail could be left (35). One or more voicemails was left for some individuals (100). Many of those contacted had lost interest due to illness, scheduling or age (52). Six interviews were cancelled or withdrawn. No contact could be established with the remainder. Of the 49 who consented and interviewed, 40 were interviewed over Zoom and 9 over the phone. All recordings were of acceptable audio quality and were transcribed for use in analysis.

The interview contained three parts, 1) the Cookie Theft task (CT) from the Boston Diagnostic Aphasia Examination (28, 29) (**Supplementary, Appendix A**). 2) three open ended questions about aging (AG) (**Supplementary, Appendix A**) and 3) two structured questionnaires, the 12 -item Modified Telephone Interview for Cognitive Status (TICS-m) (57) and the 25-item Cognitive Failures Questionnaire (CFQ) (58, 59). The interviewer was trained to administer the TICS-m and CFQ tasks by a licensed staff psychologist who was also available to answer scoring related questions. Interviews were conducted over Zoom or phone between June 2024 and December 2024 and study data were managed and collected using REDCap electronic data capture tools hosted at UC San Diego (60–62).

### 2.2 Sociodemographic and clinical neuropsychological measures

Socio Demographic: Sociodemographic information was made available from participant details in the parent survey (56) which included age, sex, race, marital-status and education.

Cognitive Failures Questionnaire (CFQ) (58) is a 25-item questionnaire of self-reported failures in perception, memory, and motor function. Responses are stable over a long period, tend to show positive correlation among questions, and positively correlated with the number of psychiatric symptoms reported on the Mental Health Quotient (MHQ). A high CFQ score was defined as greater than or equal to 43, which was associated previously with neurosarcoidosis (63).

The Modified Telephone Interview for Cognitive Status (TICS-m) (57, 64) is a concise questionnaire adapted to be used over the phone for screening dementia or mild cognitive impairment (MCI). The questions on TICS-m target attention, orientation, language, and learning and memory like the Mini-Mental Status Exam (MMSE). The modified version includes delayed recall for better detection of memory deficits compared to the original. We administered the 12 -item Telephone Interview of Cognitive Status (TICS-m) (57) which is a modified version of 11-item Telephone Interview of Cognitive Status (TICS) (65). Item 10 of TICS and TICS-m which is “With your finger tap five times on the part of the phone you speak into”, was replaced by “Clap your hands five times” where the interviewer could see or hear the participant clapping on the Zoom/phone. Two studies offer detailed comparison of various versions of TICS to assess consistency of cutoffs (66, 67). A meta-analysis recommended a cut-off score of <31 on the TICS, providing 92% sensitivity and 66% specificity for detecting dementia (68). A cut-off score of 30/31 with 85% sensitivity and 83% specificity was suggested for the TICS-m assessment (57) and goes on to suggest that cutoff of 31/32 produces similar discrimination. This is also supported by (69).

### 2.3 Audio preprocessing

The audio recordings were converted to.wav format. These recordings were used in entirety to extract acoustic features with an assumption that interviewer utterances comprised of only a small part of the recording and were consistent. Digital recordings of Zoom or phone-based interviews were obtained in.m4a or mp3 formats respectively and then converted to.wav format using *ffmpeg* (70). The audio recordings included the interviewer’s prompts which were short and generally uniform across the sample.

### 2.4 Features

Acoustic Features: We used the concise and curated feature set “eGeMAPSv02” suitable for clinical speech analysis, and described in Geneva minimalistic acoustic parameter set (GeMAPS) for voice research and affective computing (71). The GeMAPS is a minimal set of voice acoustic features that are deemed suitable for both voice (trait) and mood (state) related research (3) and made accessible through the Python openSMILE library (audEERING GmbH), and validated for this purpose (35–37) relevant among these are features of successive formants, F0, F1, and F2, the successive peaks in the frequency spectrum, and voice shimmer. Details on the acoustic features can be found in (71, 72), (**Supplementary, Appendix B**).

Psycholinguistic Features: The recordings were transcribed using whisper (https://whisperapi.com/speech-to-text-free-tool). The transcribed text was then manually tagged with “Q:” tags for interviewer utterances and “A:” for the participant utterances. These tags were used to extract participant utterances for further LIWC analysis. LIWC uses a word spotting paradigm as used in Linguistic Inquiry through Word Counting (LIWC) (43), considered to be the gold standard in NLP for psychology applications. The approach emphasizes content over syntax. The technique typically uses a handcrafted dictionary, that has assigned words to categories, to count words in the text that fall in each category, We extracted the full set of 119 LIWC 2022 features described by (43) for each transcript in our dataset. Transformer based approaches such as BERT (73) or large language models (LLMs) require large to huge amounts of data while offering little insights into the relevance of features. Further, they use only textual data and do not incorporate audio features. Finally, the performance as assessed through F1-score was low (74). Therefore, these approaches were not investigated.

Demographic Features: Age, sex, race and years of education were considered in the feature set.

### 2.5 Feature ranking

Gini ranking (75) was used to rank top 20 features. The contribution of the features in discriminating the cognitively impaired (TICS-m <= 31) from those who were not, and those with significant self-assessed cognitive complaints (CFQ >42) from those without, was assessed by first limiting the feature set to include only the top 20 features. The vast total number of features (119 linguistic features, 88 acoustic features from derived from each of the two tasks (**Supplementary Figure A1**) and 5 demographic features) far exceed the sample size (n=49), creating a strong likelihood of overfitting.

### 2.6 Machine learning models

We used ANN with ReLU, logistic and tanh activation functions, Support vector machine (SVM) and k neighbors (kNN) based models with specified hyperparameters (**Supplementary, Appendix C**). The aim was to assess features and tasks in their utility, rather than to obtain the best fine-tuned classification model. Transformer based approaches such as BERT (73) or large language models (LLMs) require larger data sets and are also limited in respect to interpretability of features. Further, they use only textual data and do not incorporate audio features. Therefore, these approaches were not applied in the current study.

Several model performance metrics were evaluated such as Area Under the Curve (AUC), F1 score, precision, recall and specificity and the details on how these measures are computed are available in the supplementary material (**Supplementary, Appendix D**). AUC provides an overall picture of model’s ability to classify beyond randomness on a range on operating points, precision and recall may provide a direct measure for comparison in specific applications (e.g. higher recall may be desirable over precision in medical screening applications). We use the harmonic mean of precision and recall, the F1-score, to rate feature-set and model performances.

## 3 Results

**Figure 2:**
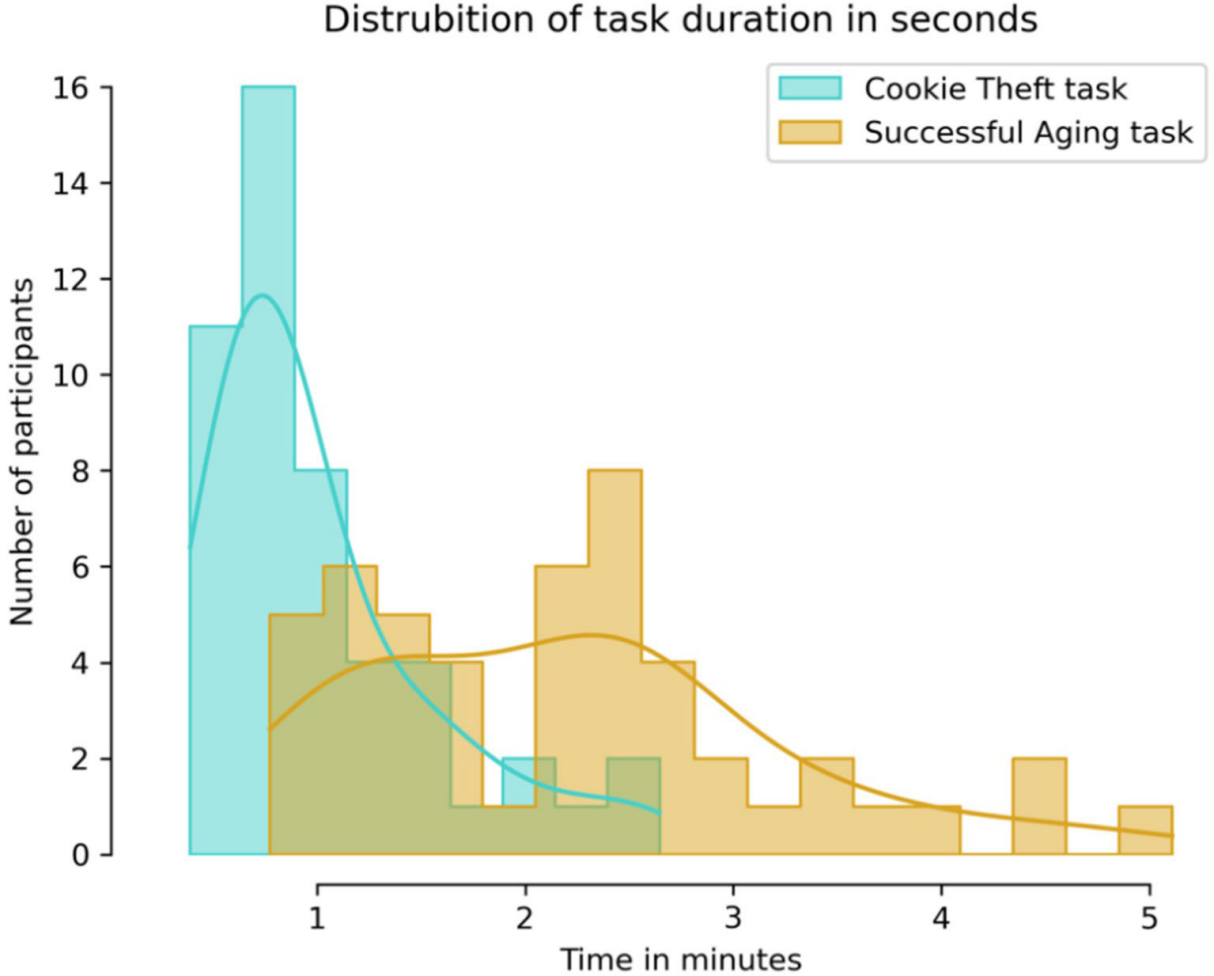
Distribution of task durations in minutes. CT task was generally completed within a minute, while AG task durations were bi-modal, approximately one half taking a little over a minute, and the other about twice as much.

**Figure 3:**
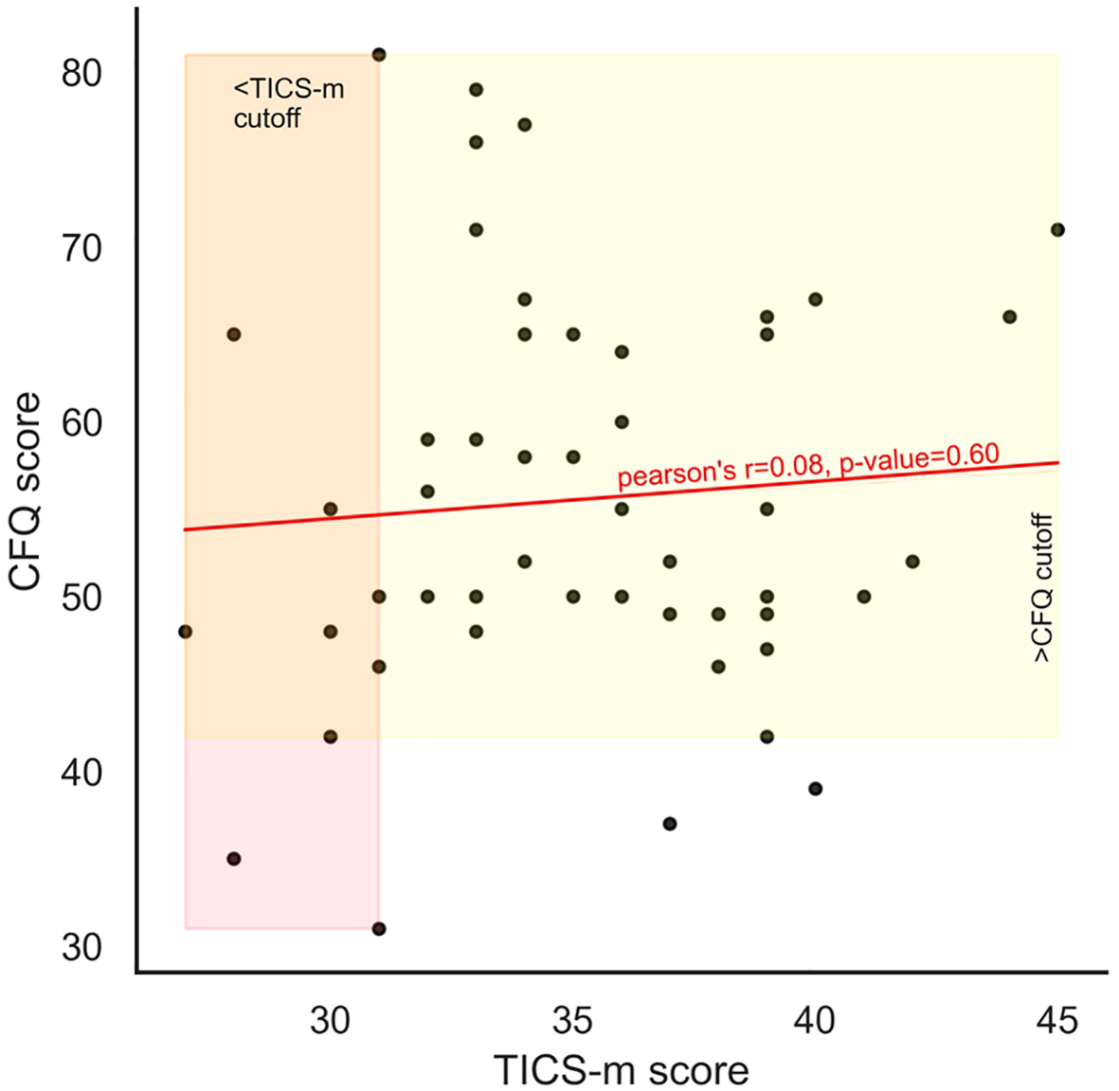
Objective cognition as measured using TICS-m and subjective cognition as measured using CFQ show no correlation.

The sample ranged from 61 to 93 years in age at the time of interviews (**Table 1**). Participants were mostly white 42(85.7%), female 26(53.1%) and married or cohabitating (n=33, 67.3%), with high education (mean years15.6, SD 2.2). Cognitive functioning varied among participants as indicated by TICS-m scores in the range 27–45 with a mean score 35.1 (SD=4.1), with 10 (or 20.4%) falling below the cutoff (<=31). Subjective failures of cognition as reported in the CFQ were in the range 31-81, with a mean score 55.5 (SD=11.5), with 43 (or 87.8%) reporting above the cutoff (>42). The Zoom recordings were compared with phone recordings and no discernible differences in quality were observed. No recording got excluded from analysis due to poor quality. The CT task duration was unimodal, lasting a minute in most cases (Mean= 1.0 minutes, SD=0.5), while the AG task duration was bimodal with one peak a little over a minute and the other about two and a half minutes (Mean=2.2 minutes. SD=1.0) (**Figure 2** **).** **Supplementary Figure B1** shows effect of computing features on longer time scales may cause some loss of momentary information. CFQ and TICS-m scores for individuals were not correlated (**Figure 3**).

**Table 1:** Demographic and clinical characteristics.

Characteristics	Specification	Mean (Std. Dev); Min-Max	N(%)
Age		76.9(8.5); 61.2-93.8	
Sex	Female		26(53.1%)
Race	White		42(85.7%)
	Hispanic		4(8.16%)
	Other		3(6.12%)
Education	Number of years	15.6(2.2); 11.0-18.0	
Marital Status	Currently Married/Cohabitating		33(67.3%)
	Never married/divorced/separated		8(16.33%)
	Widowed		7(14.29%)
	Other		1(2.04%)
CFQ score		55.5(11.5);31.0-81.0	
	CFQ > 42		43(87.8%)
TICS-m score		35.1(4.1);27.0-45.0	
	TICS-m <=31		10(20.4%)

When predicting subjective cognition based on elevated CFQ score, ML models performed best with all combined features including acoustic and content-based features were combined from CT and AG tasks. Using single-learner models an F1-score of 0.83, precision of 0.85 with an AUC of 0.88 was achieved (**Table 2A**). Similarly, when predicting objective cognition based on TICS-m cutoff, ML models performed best with all combined features including acoustic and content-based features from both CT and AG tasks. We achieved an F1-score and precision of 0.92 with an AUC of 0.90 (**Table 2A**). Performance improved for some targets when ensemble methods were used (**Table 2B**). All models and targets yielded AUC equal to or above 0.76, with TICS-m classification approaching 0.90; these values are sufficiently higher than 0.5 (random) suggesting the identified features have great value in classification. Precision, recall and their harmonic mean, the F1 score, too, support our claim of excellent classification. **Tables 3**, **4** show the best features identified through GINI-index that were used for classification.

**Table 2A:** Best performing single estimators/learners.

Target	Features	Model	AUC	F1	Precision	Recall	Specificity
CFQ	All	ANN	0.88	0.83	0.85	0.82	0.54
CFQ	AG and CT	ANN	0.86	0.82	0.83	0.82	0.40
CFQ	AG	ANN	0.76	0.82	0.83	0.82	0.40
CFQ	CT	kNN	0.80	0.82	0.77	0.88	0.12
Tics-m	All	ANN	0.90	0.92	0.92	0.92	0.83
Tics-m	AG and CT	ANN	0.90	0.90	0.90	0.90	0.82
Tics-m	AG	ANN	0.76	0.76	0.76	0.80	0.35
Tics-m	CT	ANN	0.88	0.80	0.81	0.80	0.65

**Table 2B:** Best performing models when ensemble methods were included.

Target	Features	Model	AUC	F1	Precision	Recall	Specificity
CFQ	All	AdaBoost	0.81	0.92	0.92	0.92	0.70
CFQ	AG and CT	AdaBoost	0.71	0.88	0.88	0.88	0.55
CFQ	AG	AdaBoost	0.71	0.88	0.88	0.88	0.55
CFQ	CT	Random Forest	0.80	0.89	0.89	0.89	0.56
Tics-m	All	ANN	0.90	0.92	0.92	0.92	0.83
Tics-m	AG and CT	ANN	0.90	0.90	0.90	0.90	0.82
Tics-m	AG	XGBoost	0.83	0.81	0.82	0.84	0.44
Tics-m	CT	ANN	0.88	0.80	0.81	0.80	0.65

AG- Acoustic, linguistic features related to aging related questions.

CT- Acoustic, linguistic features related to Cookie Theft picture description.

ALL- All combined features (AG, CT and Demographics).

AUC-Area under the curve.

F1-F score.

ANN-Artificial neural network, ReLu.

kNN- k nearest neighbour.

XGBoost-Gradient Boosting.

**Table 3:** Top features for predicting Cognitive Failures Questionnaire (CFQ) score > 42.

CFQ (Subjective experience of cognitive aging)						
Feature rank	Task type	Feature name - description	Information gain	Gini	Student’s t	P value
1	AG OpenSmile	**shimmerLocaldB_sma3nz_amean** – Loudness (Variation)	0.195	0.058	[+] 3.062	0.004*
2	AG OpenSmile	**spectralFluxV_sma3nz_amean**	0.195	0.058	[+] 2.973	0.005*
3	AG LIWC	**Tentat** - Tentative (E.g. if, or, any, something)	0.195	0.058	[+] 1.919	0.063
4	CT LIWC	**Tone** - Emotional tone (Degree of positive (negative) tone)	0.144	0.043	[-] 0.895	0.388
5	CT LIWC	**Comm** - Communication (E.g. said, say, tell, thank)	0.116	0.043	[-] 1.665	0.150
6	CT LIWC	**Negate** - Negations (E.g. not, no, never, nothing)	0.167	0.040	[-]1.796	0.125
7	AG LIWC	**Leisure** - (E.g. game, fun, play, party)	0.114	0.040	[-] 0.997	0.348
8	AG OpenSmile	**loudness_sma3_percentile20.0** – Loudness (Baseline)	0.152	0.038	[+] 3.084	0.003*
9	AG OpenSmile	**loudness_sma3_stddevFallingSlope** – Loudness (Rolloff)	0.152	0.038	[+] 2.810	0.009*
10	AG OpenSmile	**mfcc2_sma3_stddevNorm**	0.152	0.038	[-] 0.743	0.462
11	CT OpenSmile	**F0semitoneFrom27.5Hz_sma3nz_percentile20.0** – Formant 0 (Baseline frequency)	0.152	0.038	[+] 0.414	0.688
12	CT OpenSmile	**shimmerLocaldB_sma3nz_amean** –Loudness (Variation)	0.152	0.038	[+] 1.089	0.291
13	CT OpenSmile	**MeanVoicedSegmentLengthSec** – Voiced Speech length (mean)	0.152	0.038	[+] 0.631	0.533
14	CT LIWC	**Analytic** - Analytical thinking (Metric of logical, formal thinking)	0.152	0.038	[+] 2.578	0.026*
15	CT OpenSmile	**jitterLocal_sma3nz_amean** – Frequency (Shifts)	0.147	0.037	[-] 0.728	0.488
16	CT LIWC	**Health** - (E.g. medic, patients, physician, health)	0.101	0.034	[-] 0.129	0.900
17	AG OpenSmile	**mfcc2_sma3_amean** – 2^nd^ Mel Cepstrum (Voice Timber)	0.141	0.034	[-] 4.946	0.001*
18	AG OpenSmile	**mfcc2V_sma3nz_amean** – 2^nd^ Mel Cepstrum (Voice Timber)	0.141	0.034	[-] 0.4531	0.001*
19	AG LIWC	**Allure** - words commonly used in successful ads and persuasive communications (76).	0.141	0.034	[-] 1.200	0.267
20	CT OpenSmile	**mfcc2_sma3_amean** – 2^nd^ Mel Cepstrum (Voice Timber)	0.141	0.034	[-] 4.783	0.001*

AG: Aging questions.

CT: Cookie Theft picture description.

[+]: positively related to CFQ score > 42.

[-]: negatively related to CFQ score > 42.Feature names are in bold and a short description is provided in normal text.*: Significant p-values < .05.

**Table 4:** Top features for Modified Telephone Interview for Cognitive Status (Tics-m) score <=31.

TICS-m (Objective assessment of cognitive aging)						
Feature rank	Task type	Feature name-description	Information gain	Gini	Student’s t	P value
1	CT LIWC	**Number** - (E.g. one, two, first, once)	0.154	0.074	[+] 0.980	0.338
2	AG LIWC	**Affiliation** - (E.g. we, our, us, help)	0.183	0.073	[-] 2.311	0.032*
3	CT LIWC	**Feeling** - (E.g. feel, hard, cool, felt)	0.126	0.067	[+] 1.671	0.125
4	CT LIWC	**emo_neg** - negative emotion	0.132	0.066	[+] 0.747	0.467
5	CT OpenSmile	**F2frequency_sma3nz_amean** – Formant 2 (frequency)	0.166	0.064	[+] 1.935	0.073
6	CT OpenSmile	**F0semitoneFrom27.5Hz_sma3nz_stddevFallingSlope –** Formant 0 (rolloff variation)	0.166	0.064	[+] 2.957	0.013*
7	CT OpenSmile	**F0semitoneFrom27.5Hz_sma3nz_meanFallingSlope –** Formant 0 (rolloff mean)	0.166	0.064	[+] 2.729	0.021*
8	AG LIWC	**emo_anx** - anxiety	0.120	0.061	[+] 1.744	0.111
9	AG LIWC	**Work** - (E.g. work, school, working, class)	0.155	0.060	[-] 3.267	0.003*
10	CT OpenSmile	**slopeV0-500_sma3nz_amean**	0.164	0.060	[-] 1.600	0.134
11	CT OpenSmile	**F0semitoneFrom27.5Hz_sma3nz_pctlrange0-2** – Formant 0 (range)	0.161	0.059	[+] 0.498	0.629
12	AG OpenSmile	**F1bandwidth_sma3nz_stddevNorm** – Formant 1 (bandwidth)	0.118	0.059	[-] 0.384	0.706
13	AG LIWC	**Perception** - (E.g. in, out, up, there)	0.152	0.055	[-] 0.214	0.835
14	AG OpenSmile	**mfcc2V_sma3nz_amean -** 2^nd^ Mel Cepstrum (Voice Timber)	0.149	0.053	[+] 0.876	0.399
15	AG OpenSmile	**F3frequency_sma3nz_amean -** Formant 3 (frequency)	0.149	0.053	[+] 1.066	0.304
16	CT LIWC	**Space** - E.g. in, out, up, there)	0.132	0.046	[-] 3.105	0.005*
17	CT LIWC	**BigWords** - Percent words 7 letters or longer	0.132	0.046	[-] 2.663	0.020*
18	CT LIWC	**Allnone** - (E.g. all, no, never, always)	0.110	0.045	[+] 1.893	0.082
19	AG OpenSmile	**mfcc2_sma3_stddevNorm -** 2^nd^ Mel Cepstrum (Voice Timber variation)	0.127	0.045	[+] 0.337	0.738
20	AG LIWC	**Conj** - (E.g. and, but, so, as)	0.124	0.044	[-] 1.069	0.307

AG: Aging questions.

CT: Cookie Theft picture description.

[+]: positively related to Tics-m score <=31.

[-]: negatively related to Tics-m score <=31.Feature names are in bold and a short description is provided in normal text.*: Significant p-values < .05.

The AG and CT tasks, however, contributed differently to discrimination of elevation in subjective cognitive complaints as well as low scores on objective cognition measure. In predicting CFQ, seven of the top 10 features were derived from the AG instead of the CT task. In contrast, when predicting TICS-m scores, seven of the top 10 features were derived from the CT instead of the AG task. Further, the specificity for CFQ target was poor with features derived only from the CT task, suggesting limited suitability of the task for the CFQ classification. The generally lower specificity for the CFQ target, we believe, stems from the fact that 87.76% of our sample had CFQ above the cutoff (**Table 1**), and the models were eager to classify samples into the category. As expected, using the top ranked features from combined set yielded the best classification; F1 of 0.92 for the TICS-m target and 0.83 for the CFQ (**Table 2A**). Demographic features (age, sex, race, education and marital status) were of little consequence (**Tables 3**, **4**). CT derived features were more relevant to TICS-m classification while AG derived features were of greater value to the CFQ classification.

Importance by the type of features, openSMILE (acoustic) vs. LIWC (content) as represented among top 10, in prediction of the two targets were split evenly. When predicting CFQ based subjective cognition, five were derived from openSMILE. Of these five, three acoustic features were loudness and shimmer (changes in loudness) related (**Table 3**). The LIWC features derived from AG task encoded tentativeness and leisure in the reminiscence. The CT task derived LIWC features encoded tone and negation (**Supplementary, Figure B2**). When predicting TICS-m based objective cognition, four features were derived from openSMILE, three of which encode aspects of formant frequencies, F0 (lowest) to F2 (highest). The AG task derived LIWC features encoded work and affiliation (**Supplementary, Figure B3**). The remaining features encoded negative emotion and anxiety.

## 4 Discussion

Notwithstanding limitations, we found several potentially important aspects of different speech collection modalities and predictive accuracy across subjective and objective cognition in older adults. Our hypotheses were supported; acoustic and linguistic markers derived from either the cookie theft and open-ended modalities achieved acceptable accuracy in predicting objective and subjective cognition. However, the features derived from the cookie theft task were more predictive of objective measure of cognition, whereas the more open ended successful aging questions derived features were more predictive of subjective complaints. In all models, there was a relatively balanced proportion of acoustic versus linguistic markers in prediction of both objective and subjective cognition, with little overlap in top features across prediction of subjective or objective cognition. Therefore, different speech elicitation modalities (cookie theft, open-ended etc.) may have different strengths in predicting objective and subjective cognition, and the combination of acoustic and linguistic markers may be optimal in predicting either outcome.

Our study contributes to a growing literature evaluating the linguistic and audio features derived specifically from the cookie theft picture description task as well as other brief structured cognitive tasks (77). Our study was different from many in that included people who were randomly selected from a population and evaluated the link to task performance rather than diagnostic characterization (e.g., MCI). A focused review on cookie theft task studies suggested richness of content words, conciseness of expression, and quantity of expression were greater among the control (31). A recent review proceeded to harmonize the linguistic feature nomenclature that abounds (into several 100s) in the literature, found that linguistic feature categories such as phonetic-prosody (breaks and repetitions in connected speech), lexical-semantic (meaning and grammar), speed, coherence and cohesion were very relevant in screening (78), these reviews did not include acoustic features. We found that language suggestive of negative tone, use of numbers from the CT task, and work and affiliation from the AG task were relevant linguistic features. Acoustic features that captured formant frequencies were also discriminative. Our study evaluated the prediction of a global cognitive screening measures across domains (3), and future studies might employ a comprehensive neuropsychological battery to evaluate which acoustic and linguistic features align with different cognitive domains.

Our study is consistent with recent studies on speech analysis and objective cognition. A study (77) used audio features extracted using openSMILE and Wave2Vec2.0 (79) which is an alternative audio feature representation. The highest accuracies reported (84.8%) were from the interference and the number reading task while the interview and reading task provided lower accuracies in the 67%-78% range. While the performance of openSMILE and Wave2Vec derived features were identical for the best case of interference task. These accuracies are about 5% lower than our best performances which we attribute to their simpler model choice of Support Vector Machine (SVM), and a lack of feature selection. The feature relevance was not examined, but the study reinforces our finding on different speech elicitation modalities where features derived from tasks of cognition are better predictors of objective cognition. Accuracies like ours were achieved in Chinese language Cookie Theft task with simpler audio features that encoded pauses and hesitation but included visual facial features (80). BERT based models that used transcriptions (only) of the cookie theft task achieved lower accuracies of about 84.8% for non-controls (81), suggesting acoustic features have additional and relevant information besides transcriptions only processing by BERT, a notion also embraced by a recently proposed dementia screening system (82).

Our study was novel in evaluating and applying speech analysis to the prediction of subjective cognition. Acoustic and linguistic markers were able to predict subjective cognition. Notably, the markers were generally different from those of objective cognition, with some overlap of linguistic markers of negative emotions. Recent reviews of longitudinal studies suggest a higher symptom burden on subjective cognition has predictive value for mild cognitive impairment (MCI) and dementia (83), while the symptoms themselves were associated with quality of life (84). Conversely, a younger subjective age was related to higher cognitive performance, and reduced depressive symptoms (85) (86), suggesting subjective cognition, quality of life, subjective age, depressive symptoms and longer term cognitive outcomes remain enmeshed (87). Other studies provide evidence that subjective cognition and depressive symptomology may be directly linked as higher cognitive failure scores are associated with greater perceived psychological distress and affective disorders (13, 58, 88, 89), and momentary affect among healthy individuals (90). The association of negative emotions with subjective cognition is therefore not surprising. Subjective cognitive complaints are a component of MCI diagnoses, but a challenge is in potentially understanding the specificity of these experiences beyond affective symptoms. Furthermore, since our open-ended question likely elicited more affectively linked content, it is perhaps not surprising that open ended questions content was more linked to subjective compared to objective cognition. In the future, sentiment from NLP and audio features that encode emotions, such as shimmer, could play a role is disentangling symptoms from subjective complaints. The use of multimodal speech elicitation paradigms may help tease apart the subjective complaints tied to objective decline from that tied to affective symptoms. In the future, it would be important to understand the within person trajectories of acoustic and linguistic features and how they might change with subjective and objective cognition. Other linguistic features such as sentence complexity, vocabulary richness and attributes of grammar might be more stable and linked to crystallized knowledge, whereas features that are related to vocalization and sentiment may vary within people, and perhaps in conjunction with affective states.

Acoustic features implicated in subjective cognitive complaints in our study were shimmer, spectral flux and loudness, all derived from the AG task (among top 10, **Table 3**). In speech analysis, “shimmer” is an acoustic feature that quantifies the cycle-to-cycle variation in the amplitude (loudness) of a voice signal, as how much the loudness fluctuates between each vocal fold vibration. This is in accordance with other studies that have linked shimmer and loudness with emotions (38, 39, 91); and emotions having established link to SCC is in alignment with our previous finding that such complaints are mood dependent (90).

In contrast, key audio features implicated in objective cognition were all related to base and higher formant frequencies derived from the CT task. Formant frequencies were shown to undergo a predictable change under cognitive load (41). Such formant shifts (at a gross level) are manifested as a shift in pitch and Mel-frequency cepstral coefficients (mfcc), which was described as an invariant pattern of cognitive decline (92). There is a greater body of evidence supporting this finding (3, 93, 94). The probable explanation for fundamental frequency (F0) and resonant frequencies (Formants) to encode information about an individual’s cognition stems from the mechanics of phonological motor planning and control of vocal speech production apparatus (95). Inclusion of F0, F1, and F2 formant features in analysis of interview prompts that require cognitive processing can be helpful in assessing individual cognitive capacities and as indicators of cognition decline (40).

Our study had several strengths including the focus on multiple modes of speech elicitation, prediction of both objective and subjective cognition, and inclusion of both acoustic and linguistic markers. There are some important limitations and, as such, this study’s findings should be considered preliminary and require replication. For one, the sample size was small, and the demographic make-up of the sample was skewed toward white and persons with high education. The sampling approach employed random-digital dialing (56) but we note that this is a subset of the original sample. Our study’s outcomes included brief global screenings of cognitive ability and subjective cognition and so does not speak to the prediction of specific cognitive impairments or diagnoses (e.g., MCI). There is a myriad of potential prompts for elicitation of speech. In the future, data from a larger more diverse population (or integrable data sets from different populations) alongside a wider variety of prompts that are parameterized for variation in subjective or objective cognitive levels derived from normed data would help to specify prompts that produce audio and linguistic patterns linked to either subjective, objective cognition or both. The study is also cross sectional and does not speak to the stability of these findings, and did not include independent validation. In our current survey we did not have questions about subjective cognition from the perspective of caregivers. As such replication would be required to understand the robustness of these findings. Finally, NLP models used supervised approaches and generative or transformer models could provide additional accuracy.

As a basis for future work, next steps would include replication in larger sample and designing of prompts that elicit content predictive of objective versus subjective cognition. It would be helpful also to contrast people with and without objective cognitive impairments on the acoustic and linguistic predictors of subjective complaints. Further, the influence of mood and other factors on the stability of speech features would be useful, in particular via longitudinal study that might evaluate MCI conversion as an endpoint. A larger study sample on casual conversation would facilitate topic-modelling and clustering approaches for thematic analysis. Furthermore, understanding how speech markers evolve over time in concert with subjective and objective cognitive, as well as brain and other biological markers, would be highly informative. Analyzing such conversations using LLMs with ingrained reasoning and BERT based classification are natural next steps. Together, these findings are consistent with recent reviews indicating the protentional for speech analysis in understanding cognitive aging, with our study indicating that this also extends to subjective cognitive decline.

## Data Availability

The study/data is governed by University of California San Diego Human Research Protections Program (HRPP) rules and other contract. Clinical data or the code is not publicly available due to privacy concerns, including HIPAA regulations. For machine learning parameters access, qualified researchers may contact the corresponding author. Requests to access the datasets should be directed to vbadal@health.ucsd.edu.
