# Supplementary material for: Audio and linguistic prediction of objective and subjective cognition in older adults: what is the role of different prompts?
Source: Front Psychiatry. 2025 Jul 1;16:1596132. doi: 10.3389/fpsyt.2025.1596132 (PMC12259555; doi:10.3389/fpsyt.2025.1596132)
Supplement: Supplementary file 1 [file SupplementaryFile1.docx]

**Appendix A:** **Qualitative questions in the semi-structured interviews that was analyzed in this project.**

| a) | The Picture used for the cookie Theft task is available from the original publication from the following links  https://www.jbe-platform.com/content/journals/10.1075/ps.17011.cum#dataandmedia |
| --- | --- |
| b) | 1. “How do you define successful aging? 2. What is important to aging successfully? 3. Do you consider yourself to be aging successfully? Why or why not?” |

**Figure A1. Unstructured prompts**

**a) The Cookie Theft task from the Boston Diagnostic Aphasia Examination.** The cookie theft picture (Cummings, 2019; Goodglass & Kaplan, 1983) is a black-and-white line drawing of a women wiping a dish near an overflowing sink in a kitchen with an open window. Two children are shown in the background, the boy on a toppling stool reaching for a cookie from a cookie jar while a girl seem to be asking for one. **b) *Three open ended questions about aging.***

**Appendix B: Audio Feature durational stability, and distribution of features across classes**

**Changes in mean and standard deviation of a sample feature when computed over different time durations from the beginning of an audio file.**


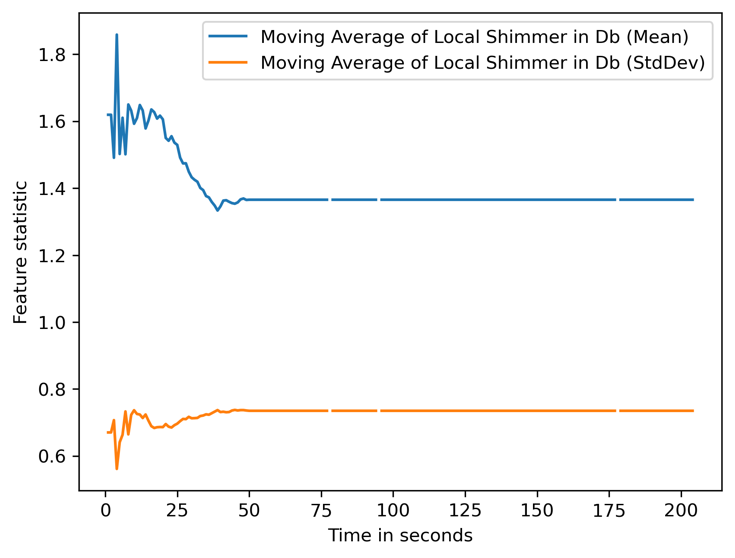

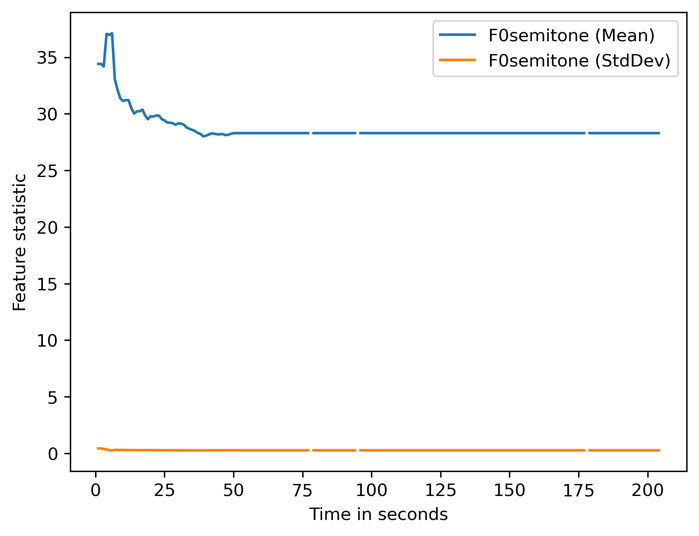


**Figure B1:** Stability of representative audio features over time. The feature means and standard deviation were computed over different lengths of audio from the same audio recording file. Short term fluctuations are lost in audio over 1 minute long, hiding momentary effects in shimmer and base formant frequencies.


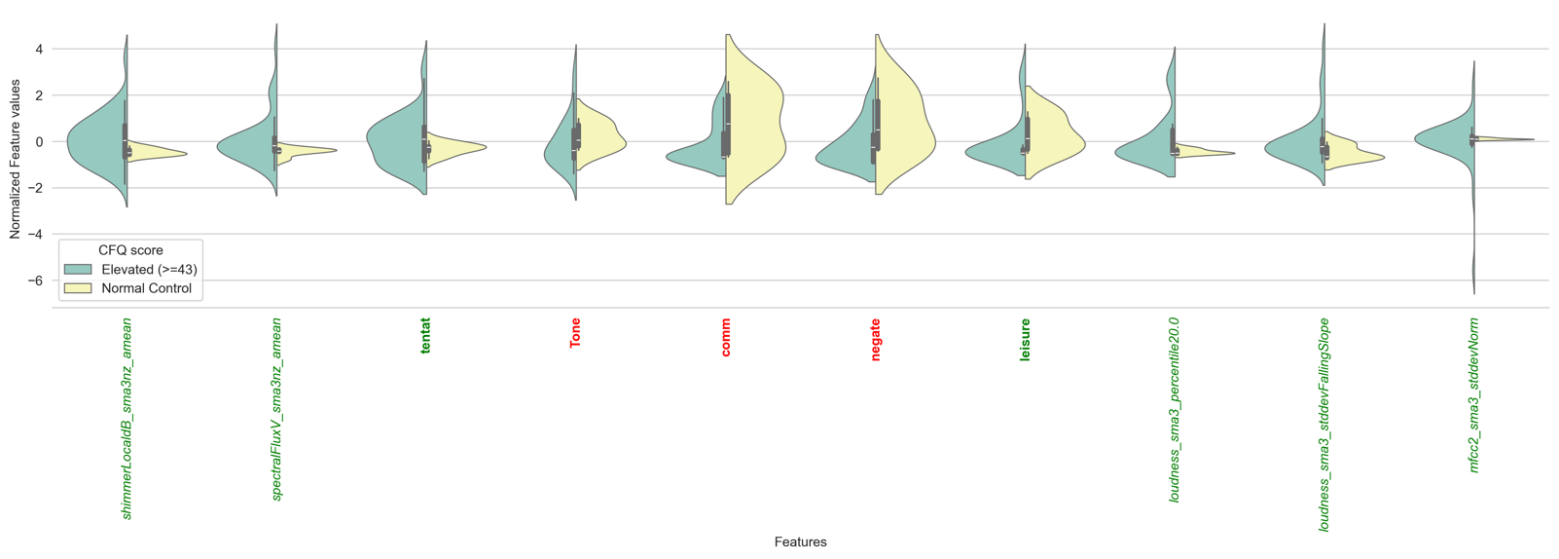


**Figure B2**: Distribution of top 10 features over classification classes for CFQ score with a cutoff >42. Audio features retain OpenSmile names for easy reference and are in *italics*. LIWC derived content features are in bold. Features originating from CT task are in red, while those from AG task are in green. For description, please see Table 3.


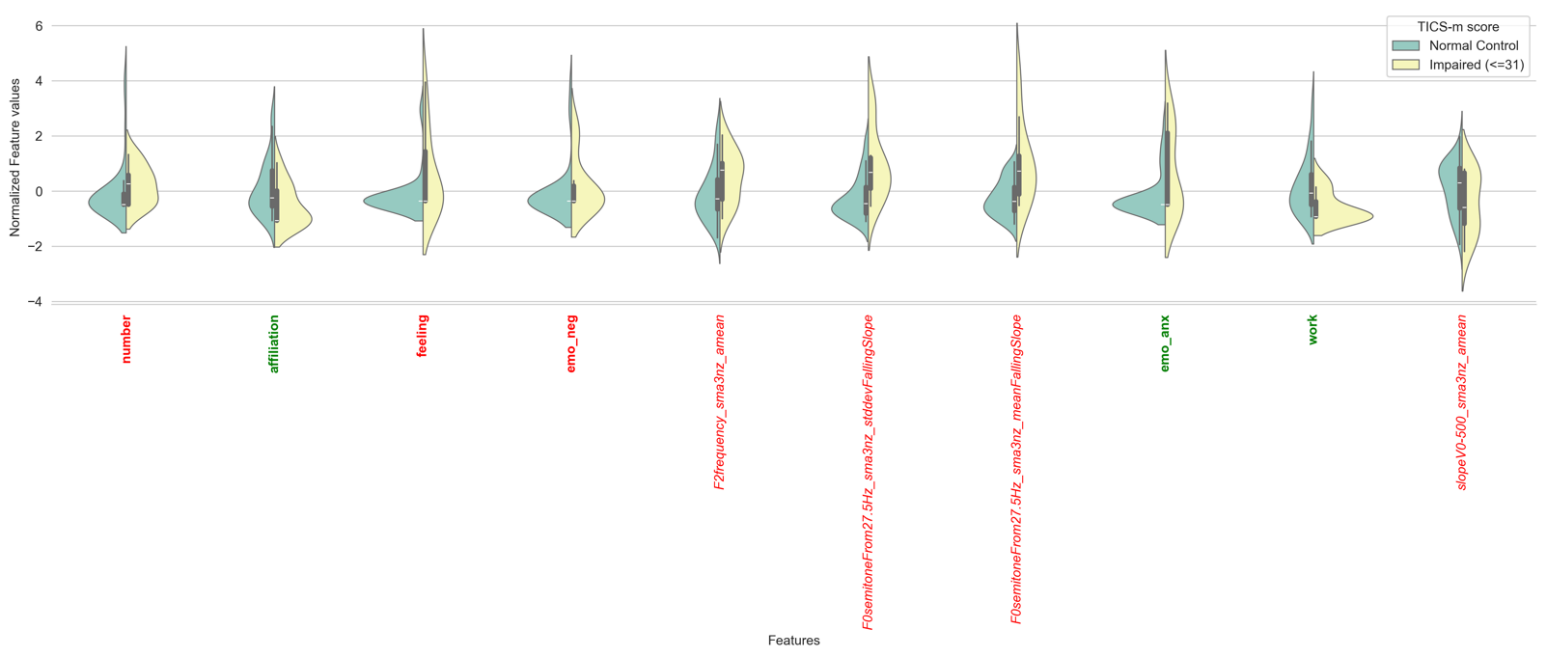


**Figure B3**: Distribution of top 10 features over classification classes for TICS-m score with a cutoff <=31. Audio features retain OpenSmile names for easy reference and are in *italics*. LIWC derived content features are in bold. Features originating from CT task are in red, while those from AG task are in green. Impaired class shows greater variance and mean (except in work and affiliation). For description, please see Table 4.

**Appendix C:** **Machine learning parameters (Demsar J, 2013).**

| **ML Model** | **Hyper parameters** |
| --- | --- |
| ANN tanh | Scipy implementation, Number of neurons in hidden layer = 200, solver=Adam |
| kNN | Number of neighjbors k = 9, Metric=Chebyshev, weight=distance |
| ANN Logistic | Scipy implementation, Number of neurons in hidden layer = 200, solver=Adam |
| SVM RBF | Cost (C)=1, Numerical Tolerance = 0.001, Epsilon=0.10, g=auto |
| ANN ReLu | Scipy implementation, Number of neurons in hidden layer = 200, solver=Adam |
| Random Forest | Number of trees = 8, Number of attributes considered at each split = 4, Limit depth of individual tree = 7, Do not spilt subsets smaller than = 2 |
| Naïve Bayes | Apply automatically |
| XGBoost | Method = Extreme Gradient Boosting (xgboost), Number of trees = 100, Learning rate = 0.300, Limit depth of individual trees = 6 |
| AdaBoost | Base estimator: Tree, Number of estimators = 50, Learning Rate: 1.0, Classification Algorithm = SAMME R., Regression Loss Function = Linear |

ANN: Artificial Neural Network

kNN: k nearest neighbors

SVM: Support Vector Machine

RBF: Radial Basis Function

ReLu: Rectified Linear Unit

XGBoost-Gradient Boosting

**Assessing model performance:**

F1 score is computed as a harmonic mean of recall and precision and hence accounts for both false positives and false negatives and works well when the classes are imbalanced.

$$F1=2(\frac{precision*recall}{precision+recall})$$

###### **Feature ranking for the best-fit ML models**

###### **GINI Index**: Gini impurity or Gini index is a popular method used in ranking the features in the ML. It represents the probability (0 to 1) that the feature is wrongly classified (0 = “pure”, 0.5 = equal distribution across all classifications, 1 = random distribution across classes) (S.D. Brown, 2009; Tyagi).

**Appendix D: Sensitivity Analysis**

| 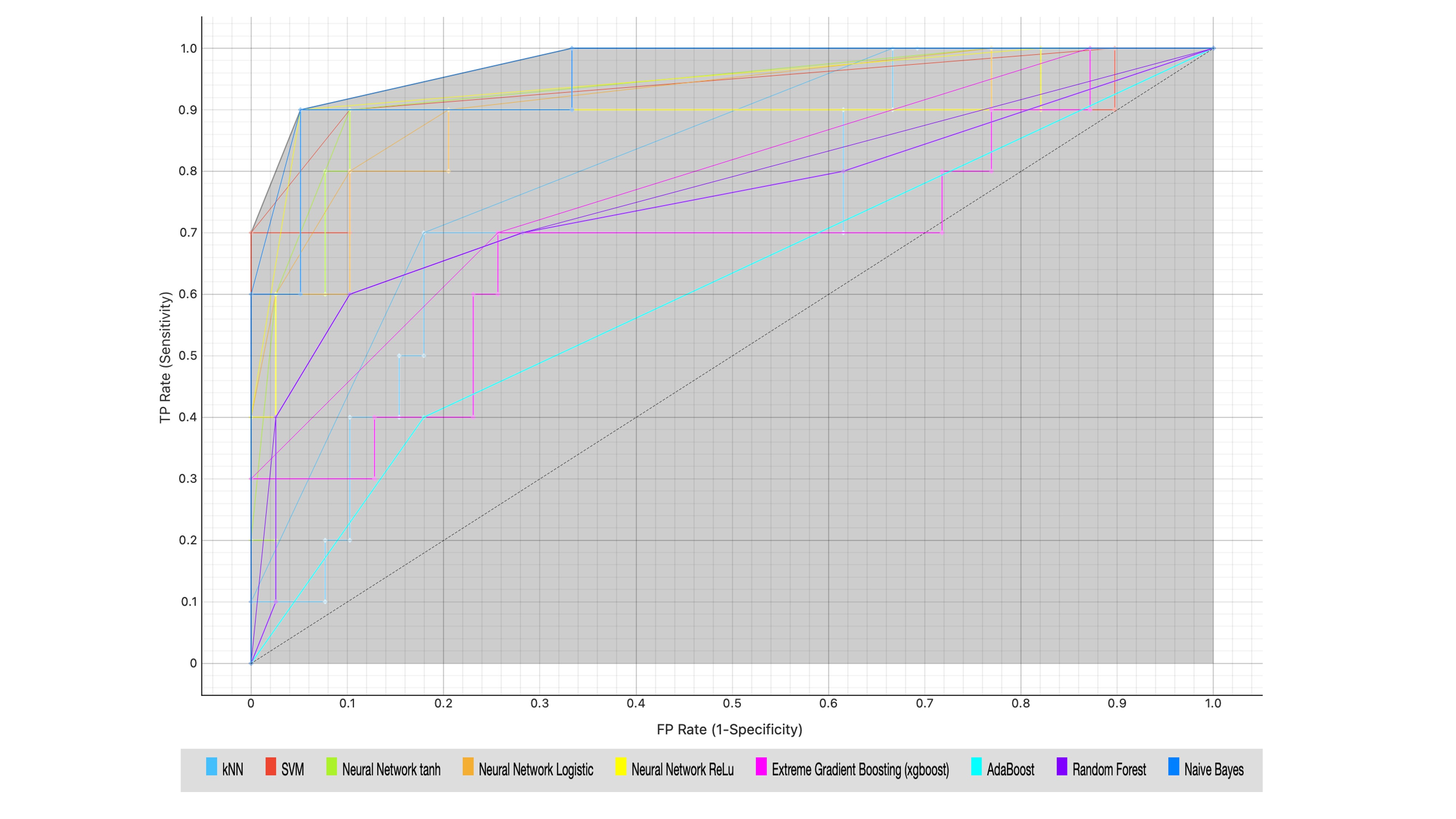 |
| --- |
| 1. Sensitivity analysis for Modified Telephone Interview for Cognitive Status (Tics-m) target (Top 20 features): Receiver Operating Characteristic (ROC) that shows sensitivity versus specificity tradeoffs for various models. |

| 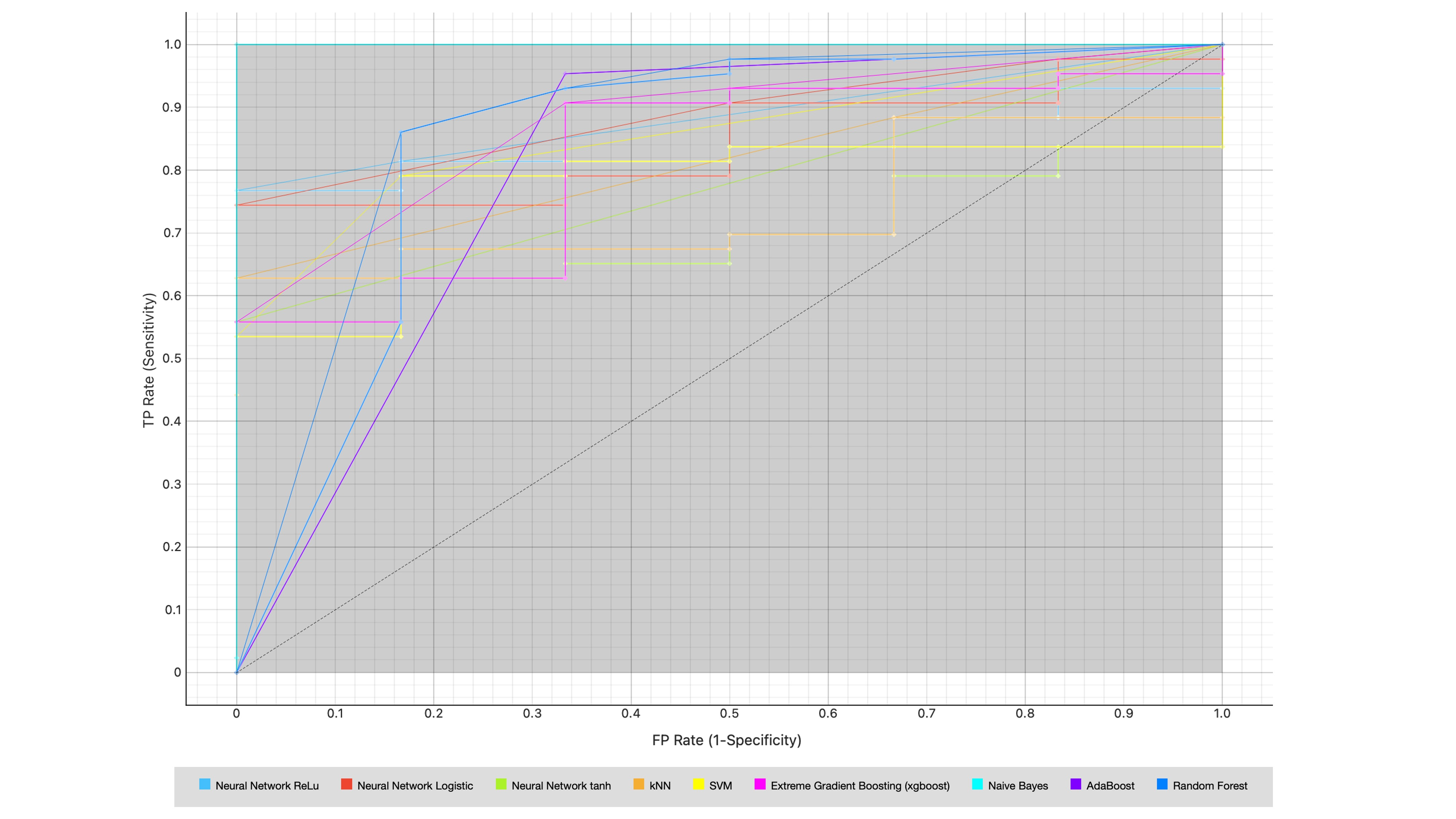 |
| --- |
| 1. Sensitivity analysis for Cognitive Failures Questionnaire (CFQ) score target (Top 20 features): Receiver Operating Characteristic (ROC) that shows sensitivity versus specificity tradeoffs for various models. |

**References**

Cummings, L. (2019). Describing the cookie theft picture: Sources of breakdown in Alzheimer’s dementia. *Pragmatics and Society, 10*(2), 153-176.

Demsar J, C. T., Erjavec A, Gorup C, Hocevar T, Milutinovic M, Mozina M, Polajnar M, Toplak M, Staric A, Stajdohar M, Umek L, Zagar L, Zbontar J, Zitnik M, Zupan B. (2013). Orange: data mining toolbox in Python. *The Journal of Machine Learning Research  14 (Aug)*, 2349-2353.

Goodglass, H., & Kaplan, E. (1983). The assessment of aphasia and related disorders. *(No Title)*.

S.D. Brown, A. J. M. (2009). <https://www.sciencedirect.com/topics/mathematics/gini-index> *Comprehensive Chemometrics , Volume 3* (Vol. 2020, pp. GINI Index). <https://www.sciencedirect.com/topics/mathematics/gini-index>.

Tyagi, N. <https://medium.com/analytics-steps/understanding-the-gini-index-and-information-gain-in-decWoSon-trees-ab4720518ba8>. *Understanding the Gini Index and Information Gain in Decision Trees*, July 2020, from <https://medium.com/analytics-steps/understanding-the-gini-index-and-information-gain-in-decision-trees-ab4720518ba8>
